# Marked Improvement in Pulmonary Arterial Hypertension in 3 Patients with a History of Amphetamine-like Drug use and Comorbidities

**DOI:** 10.2174/0118743064473701260415041740

**Published:** 2026-04-17

**Authors:** Emily King, Abraham Rothman

**Affiliations:** 1 Department of Psychology, University of California, Berkeley, CA, USA; 2 Children’s Heart Center, Las Vegas, NV, USA; 3 Division of Pediatric Cardiology, Department of Pediatrics, Kirk Kerkorian School of Medicine at UNLV, Las Vegas, NV, USA

**Keywords:** Pulmonary hypertension, Amphetamine-like drugs, Catheterization, Obesity, Xenadrine

## Abstract

**Introduction/Background:**

The prognosis of patients with Pulmonary Hypertension (PAH) associated with the intake of anorexigens and amphetamines has not been clearly defined. Published data are suboptimal due to a scarcity of corroborating hemodynamic information. There are reports of worse, the same, and better prognoses compared to patients with idiopathic pulmonary arterial hypertension.

**Case Presentation:**

Our experience with three patients who had PAH, a history of amphetamine-like drug use, and comorbidities is described, and the pertinent literature is summarized.

**Conclusion:**

Three patients with PAH and a history of amphetamine-like drug use demonstrated markedly improved hemodynamics at follow-up catheterizations.

## 1 INTRODUCTION

Amphetamine-like drugs and amphetamine abuse have been associated with Pulmonary Arterial Hypertension (PAH) [1, 2]. Fenfluramine and dexfenfluramine are methamphetamine-like drugs that have been linked to the development of PAH [3]. Patients with amphetamine-associated PAH are generally lumped together with the entire cohort of PAH patients in large studies; therefore, their prognosis has been incompletely defined. Three patients with PAH, a history of amphetamine-like drug use and comorbidities, and catheterization-proven near-normalization of pulmonary arterial pressures are described.

A literature review was performed using the search engines PubMed, Google Scholar, ScienceDirect, publisher archives (*e.g.*, Elsevier, SpringerLink), and ChatGPT. The following terms were used in the search: pulmonary hypertension and amphetamines, methamphetamines, amphetamine-like drugs, appetite suppressants, anore-xigens, drug-induced, diet pills, and follow-up catheteri-zation data. Candidate citations were identified by ChatGPT and were later verified in PubMed. No early date limit was set on the search. The search period extended through October 2025. Decisions regarding which articles to include in the paper and the table were made by author AR. Comparative cohorts reporting survival or clinical worsening in drug-associated PAH were included.

Over 40 articles were reviewed, of which 17 studies were included in the summary. Of these, 6 included catheterization-confirmed hemodynamic data from follow-up. Articles with more than 3 patients and/or good hemodynamic data were included.

Informed consent for the publication of patient infor-mation was obtained from the patients. There are molecular structural similarities for amphetamines, methampheta-mines, fenfluramine, and dexfenfluramine; therefore, in this manuscript, they are referred to as “amphetamine-like” drugs.

## 2 CASE REPORTS

### 2.1 Patient 1

A 38-year-old woman with obesity and obstructive sleep apnea had a history of Xenadrine (a combination of ephedra, caffeine, and synergine) use for 1 month in 2002 and Adipex (phentermine) use for 3 weeks in 2006. She became very short of breath after Adipex. In 2009, a cardiac catheterization revealed systemic level pulmonary arterial pressure and a good vasodilator testing response (Table **1**). Pulmonary function testing was unremarkable; she had no thromboembolic disease, connective tissue disease testing was negative, HIV status was negative. She was treated with nifedipine and then transitioned to sildenafil and ambrisentan. She had CPAP therapy for sleep apnea from 2013 to 2022. After her third catheterization (in 2021), selexipag was added to her medication regimen. She also had bariatric surgery (later in the year 2021) with a weight loss of 130 pounds. The next year, 2022, a catheterization showed a mean pulmonary arterial pressure of 20 mmHg. In 2024, she was WHO functional class I; she walked 390 meters during a 6-minute walk test, and her BNP level was 10 pg/mL. She continued on sildenafil, ambrisentan, and selexipag.

### 2.2 Patient 2

A 60-year-old woman had a history of obesity, cigarette smoking, sleep apnea, phentermine-fenfluramine use for six months in 1996, and methamphetamine abuse from 1997 to 2003. In 2003, she had a cardiac catheterization at another institution (Table **1**), after which she was started on IV epoprostenol. Pulmonary function testing was unremarkable, a CTA excluded thromboembolic disease, connective tissue disease testing was negative, and HIV status was negative. Four months later, she developed a line infection; the epoprostenol was discontinued, and she was started on bosentan. She had CPAP therapy from 2003 until 2019, and restarted in 2022. In 2005, a cardiac catheterization showed a mean pulmonary arterial pressure of 52 mmHg and a good vasodilator response. She was continued on bosentan. Two catheterizations, 6 and 10 years later, respectively, showed improved hemodynamics. Twenty years later (in 2025), a catheterization showed a mean pulmonary arterial pressure of 23 mmHg. In 2025, she was WHO functional class I; she walked 326 meters on a 6-minute walk test, and her BNP level was 42 pg/mL. She continued on bosentan therapy.

### 2.3 Patient 3

A 54-year-old overweight woman with a longstanding history of methamphetamine, mushroom, and alcohol abuse had a cardiac catheterization at another institution at 44 years of age, demonstrating a mean pulmonary arterial pressure of 60 mmHg (Table **1**). She had no response to vasodilator testing. She had no sleep apnea, pulmonary function testing was unremarkable, she had no thromboembolic disease, liver disease, or portal hypertension, and her HIV status was negative. She was treated with sildenafil and macitentan. Genetic testing revealed a TBX4 mutation. She stopped taking methamphetamines in 2023. A cardiac catheterization in late 2024, 10 years after the initial one, showed a mean pulmonary arterial pressure of 21 mmHg. She was WHO functional class I. She continued taking sildenafil and macitentan.

## 3 LITERATURE REVIEW

Table **2** summarizes reports that describe worse, similar, or better prognoses in patients with PAH associated with methamphetamine-like drugs compared with patients who have PAH of different etiologies (mostly idiopathic).

Among those reporting worse survival for patients with amphetamine-associated PAH, Rich *et al*. [4] compared 10 patients with fenfluramine-associated PAH and 70 patients with primary PAH, all treated with epoprostenol. Median survival was 14 months in the fenfluramine group, compared with 49 months in the primary PAH group. Zamanian *et al*. [5] studied 90 patients with methamphetamine-associated PAH and 97 patients with idiopathic PAH. Patients with methamphetamine-associated PAH had a higher incidence of heart failure symptoms, higher right atrial pressure, lower stroke volume, and more than double the risk of clinical worsening or death.

Others have described similar survival for patients with fenfluramine-associated PAH *vs* idiopathic PAH. Souza *et al*. [6] studied 109 patients with fenfluramine-associated PAH, some of whom also used amphetamines. The acute response to vasodilator testing was 8.3%, BMPR2 mutations were present in 22%, and the median survival was 6.4 years, similar to that of patients with idiopathic PAH. Brenot *et al*. [1] reported on 73 patients with PAH treated with epoprostenol; 15 (20%) had a history of fenfluramine use, 10 of whom had a positive vasodilatory response. Survival was similar in the fenfluramine *vs* non-fenfluramine groups. Three of the 15 fenfluramine PAH patients showed short-term clinical or hemodynamic improvement. One had a drop in pulmonary vascular resistance from 35 to 15 Wood units at 12 months. The second patient had a drop in pulmonary vascular resistance from 24 to 10 Wood units at 6 months but died 2 years later. The third patient had a decrease in pulmonary vascular resistance from 19 to 10 Wood units at 3 months, but had evidence of moderate pulmonary hypertension 4 years later. Simmoneau *et al*. [7] reported on 62 patients treated with fenfluramine derivatives (17 also used amphetamines) and 125 control patients with primary PAH. Survival was similar for both groups, 50% at 3 years. Uhland *et al*. [8] treated 64 patients with methamphetamine-associated PAH and 74 patients with IPAH with subcutaneous treprostinil. Transplant-free survival was similar: 84% at a mean follow-up of 46 months for the methamphetamine PAH group and 72.9% at a mean follow-up of 67 months for the IPAH group. Kolaitis *et al*. [9] reported data from the US-based Pulmonary Hypertension Association Registry, comparing 118 patients with methamphetamine-associated PAH and 423 patients with IPAH. Survival was similar in the 2 groups. O’Neill *et al*. [10] reported on 51 patients with methamphetamine-associated PAH *versus* 51 patients with IPAH; survival was similar at 5 years. Mlczoch *et al*. [11] described no difference in survival in 75 patients with and without anorectic drug use; none of the patients had normalization of pulmonary arterial pressure. Haddad *et al*. [12] found no difference in survival between 44 patients with IPAH and 51 patients with drug- or toxin-induced PAH.

A limited number of publications describe a better prognosis or improvement in catheterization-confirmed hemodynamics in patients with PAH associated with anorexigen drug use compared to PAH of other causes. Loogen *et al*. [13] reported on 24 patients with primary PAH and 18 patients with PAH and a history of anorexigen drug use. The 10-year survival rates were 0.31 *versus* 0.63, respectively. The mean pulmonary artery pressure increased in the primary PAH group from 48.8 to 61.0 mmHg, while it decreased in the anorexigen PAH group from 47.6 to 33.3 mmHg. Douglas *et al*. [14] described 2 patients with PAH related to previous fenfluramine use. One patient showed a decrease in mean pulmonary artery pressure from 32 to 11 mmHg over one year. The second patient experienced a decrease from 37 to 30 mmHg after 34 weeks. Pouwels *et al*. [15] reported one patient who previously used fenfluramine for 11 months. Over a three-month period, the mean pulmonary arterial pressure decreased from 47 to 16 mmHg.

## 4 DISCUSSION

Drug-induced pulmonary hypertension is rare, accounting for 10% of cases with PAH [16]. Aminorex, fenfluramine, dexfenfluramine, phentermine, and amphetamine-like drugs have been associated with the development of pulmonary arterial hypertension [1-3, 5, 16]. There are molecular structural similarities between amphetamines, methamphetamines, fenfluramine, and dexfenfluramine [17]. Several patients who take anorexigens also use methamphetamines [6, 7]. Therefore, the literature on pulmonary hypertension associated with both amphetamines and anorexigens (collectively referred to as “amphetamine-like”) was reviewed. Few publications have reviewed the prognosis of patients who have PAH associated with amphetamine-like substances, often with contradictory results and a scarcity of follow-up hemodynamic data. In comparison to idiopathic or other secondary PAH, the prognosis of patients with amphetamine-like drug use/abuse has been reported to be worse [4, 5], the same [1, 6-12], or better [13-15].

Catheterization data on three patients with pulmonary hypertension and a history of anorexigens or amphetamine-like drug use are described. Two of the 3 patients had sleep apnea and long-term CPAP therapy. The same 2 patients were responders to vasodilator testing. Long-term therapy in the 3 patients included: nifedipine, sildenafil, ambrisentan, and selexipag in patient 1, bosentan in patient 2, and sildenafil and macitentan in patient 3. All 3 patients were overweight. One of the patients experienced significant weight loss following bariatric surgery. At that time, her hemodynamics had already shown substantial improvement. At the most recent catheterization in the 3 patients, the mean pulmonary arterial pressure was 20, 23, and 21 mmHg, and the unindexed pulmonary vascular resistance was 0.9, 1.7, and 1.9 Wood units, respectively. Therefore, using contemporary PAH thresholds [18], the patients had borderline elevated pulmonary arterial pressure but normal pulmonary vascular resistance.

This manuscript’s main strength lies in the longitudinal invasive hemodynamic follow-up spanning many years, including serial right heart catheterizations and vasoreactivity testing in two patients, culminating in mean pulmonary artery pressures in a near-normal range. Such extended catheter-based follow-up is uncommon in published case literature. A second strength is the attempt to synthesize discrepant prognostic findings across studies that have reported worse, similar, or better outcomes in drug-associated PAH compared with idiopathic PAH, and to highlight the scarcity of repeated hemodynamic measurements in many cohorts.

This case series has several limitations. Two of the 3 patients had sleep apnea and were treated with long-term CPAP. It is possible that the CPAP contributed significantly to their improved hemodynamics. However, most reports of pulmonary hypertension associated with sleep apnea show only mild elevation of pulmonary arterial pressures as well as modest improvements in pressures with CPAP therapy [19]. Only three patients who demonstrated catheterization-proven hemodynamic improvement are described, and the total number of patients with similar etiology of PAH from which these cases were selected is not available. It is estimated that approximately two to three additional patients with amphetamine-associated PAH were evaluated and treated at the institution during the same timeframe. One of these patients showed echocardiographic normalization of pulmonary arterial pressure, as indicated by tricuspid regurgitation jet velocity, but was not included in this series due to refusal of catheterization secondary to lack of insurance coverage. The remaining patients have not undergone serial catheterizations. Therefore, the clinical trajectories of the 3 cases presented in this paper may still not be typical or clearly representative of methamphetamine-associated PAH. Additionally, 2 of the 3 patients were vasoreactive, in which case a hemodynamic improvement with therapy would be expected. However, whether this is solely related to the drug-associated PAH or these patients may have a different etiology for the PAH (such as sleep apnea) and were in a favorable prognostic group, cannot be discerned. Only one of the 3 patients underwent genetic testing (patient 3 was positive for a TBX4 mutation), and the effects of the genetic substrate in this patient and others remain to be determined. Evans *et al*. showed a worse prognosis in patients with BMPR2 mutations compared with patients with idiopathic PAH [20]. In Table **2**, the studies were pooled; however, they represent different eras, treatment modalities, and endpoints and may not be directly comparable. While the use of fenfluramine has decreased in the population (now used mainly for seizure control and at lower doses), the illicit and licit use of methamphetamine and amphetamine has increased significantly in the last 2 decades.

## CONCLUSION

In summary, there are limited and somewhat contradictory data on the long-term prognosis of patients with amphetamine-associated pulmonary hypertension. Most publications suggest that the survival of amphetamine-like drug-associated PAH is similar to that of idiopathic PAH. Hemodynamic data documenting the progress of patients are, however, scarce. Three patients with PAH, a history of amphetamine-like drug use and comorbidities (sleep apnea in two and a genetic mutation in one), and catheterization-proven near-normalization of pulmonary arterial pressure are described. The hemodynamic improvement may represent a treatment-associated course, the treatment of additional comorbidities in 2 of them, or possibly an intrinsically better natural history in selected patients. Hemodynamic data from more patients are necessary to make more definitive statements about physiologic evolution, the optimal choice of specific pulmonary hypertension medications, and prognosis in cases of the different amphetamine or amphetamine-like drug-associated forms of PAH.

## Figures and Tables

**Table 1:** Hemodynamics in 3 patients with presumed amphetamine and amphetamine-like-associated PAH.

**Pt**	**Date**	**RAp**	**PAp**	**mPAp**	**PCWp**	**AOp**	**mAOp**	**CI**	**PVR**	**PVR/SVR**
**1**	2009	13	80/35	52	12	82/40	65	2.8	14.3	0.77
	NO/O_2_	-	-	29	-	-	-	-	5.3	0.27
	2017	12	50/24	34	14	122/73	92	2.9	6.9	0.25
	NO/O_2_	-	-	27	-	-	-	-	2.9	0.12
	2021	13	35/18	27	15	80/51	66	2.7	4.4	0.22
	NO/O_2_	-	-	27	-	-	-	-	4.0	0.19
	2022	11	27/14	20	13	94/55	73	4.0	1.8	0.12
**2**	2003*	-	70/32	42	15	-	-	-	-	-
	2005	6	87/33	52	14	140/72	95	2.5	15.2	0.41
	NO/O_2_	-	-	32	-	-	-	-	6.0	0.18
	2011*	7	-	55	9	-	-	-	6.3	-
	2015	8	40/16	27	11	97/54	71	3.6	4.4	0.25
	NO/O_2_	-	-	23	-	-	-	-	2.7	0.16
	2025	7	35/11	23	11	112/54	78	3.6	3.5	0.18
**3**	2015*	11	88/43	60	-	130/95	-	-	16.0	-
	2024	8	29/13	21	10	87/56	70	2.9	3.8	0.18

**Note:** AOp = Aortic pressure, CI = Cardiac Index, mAOp = mean Aortic Pressure, mPAp = mean Pulmonary Artery pressure, PAp = Pulmonary Artery pressure, PCWp = Pulmonary Capillary Wedge Pressure, Pt = patient, PVR = Pulmonary Vascular Resistance indexed, PVR/SVR = Pulmonary to Systemic Vascular Resistance ratio, RAp = Right Atrial pressure. * = catheterization performed at another institution.

**Table 2:** Reports comparing the prognosis of PAH associated with amphetamine-like drug use and other forms of PAH.

**Prognosis**	**Author/Refs.**	**Number of Patients**	**Comments**
**Worse**	Rich *et al.* [4]	10 Fen	Median survival: Fen = 14 months Primary = 49 months
		70 Primary PAH	

	Zamanian *et al.* [5]	90 Meth 97 IPAH	More than double the risk of clinical worsening and death in Meth pts
**Similar**	Souza *et al.* [6]	109 Fen IPAH controls	Median survival 6.4 years. Same for both groups
	Simmoneau *et al.* [7]	62 Fen (17 also Meth)	3-year similar survival at ~ 50%
		125 controls	
	Brenot *et al.* [1]	15 Fen 58 non-Fen	similar survival
	Uhland *et al.* [8]	64 Meth 74 IPAH	similar survival
	Kolaitis *et al.* [9]	118 Meth 423 IPAH	similar survival
	O’Neill *et al.* [10]	51 Meth 51 IPAH	similar survival at 5 years
**Better**	Loogen *et al.* [13]	18 anorexigen	10-year survival: 0.63 anorexigen 0.31 Primary PAH
		24 Primary PAH	


**Abbreviation:** Fen = fenfluramine, IPAH = Idiopathic PAH, Meth = methamphetamines, PAH = Pulmonary Arterial Hypertension.

## Data Availability

The data of current study are available from corresponding author, [A.R], on a reasonable request.

## ABBREVIATION

PAH: Pulmonary Arterial Hypertension

## STANDARDS OF REPORTING

CARE guidelines were followed.
